# Alpha-Fetoprotein Response after First Transarterial Chemoembolization (TACE) and Complete Pathologic Response in Patients with Hepatocellular Cancer

**DOI:** 10.3390/cancers15153962

**Published:** 2023-08-04

**Authors:** Łukasz Masior, Maciej Krasnodębski, Mikołaj Kuncewicz, Kacper Karaban, Igor Jaszczyszyn, Emilia Kruk, Milena Małecka-Giełdowska, Krzysztof Korzeniowski, Wojciech Figiel, Marek Krawczyk, Tadeusz Wróblewski, Michał Grąt

**Affiliations:** 1 Department of General, Transplant and Liver Surgery, Medical University of Warsaw, 02-097 Warsaw, Poland; maciej.krasnodebski@wum.edu.pl (M.K.); mik.kuncewicz@gmail.com (M.K.); k.karaban99@gmail.com (K.K.); jaszczyszynigor@gmail.com (I.J.); emiliakruk1@gmail.com (E.K.); wojciech.figiel@wum.edu.pl (W.F.); marek.krawczyk@wum.edu.pl (M.K.); wroblewskitad@tlen.pl (T.W.); michal.grat@gmail.com (M.G.); 2 Department of Laboratory Medicine, Medical University of Warsaw, 02-097 Warsaw, Poland; milena.malecka@wum.edu.pl; 3 Second Department of Radiology, Medical University of Warsaw, 02-097 Warsaw, Poland; krzysztof.korzeniowski@uckwum.pl

**Keywords:** hepatocellular carcinoma, liver transplantation, alpha-fetoprotein, transarterial chemoembolization, outcomes

## Abstract

**Simple Summary:**

Transarterial chemoembolization (TACE) is the most common locoregional therapy (LRT) applied to liver transplant candidates with hepatocellular carcinoma (HCC) before liver transplantation (LT). Complete pathologic response (CPR) after LRT may be obtained in 25% of patients, which translates into better long-term results. The aim of this study was to assess the role of AFP changes after the first TACE in the prediction of complete tumor necrosis in patients with HCC. It was a retrospective, single-center study comprising 101 patients who underwent TACE before LT. Based on the initial AFP concentration and AFP decline after the first treatment, a simple scoring system, which distinguished between groups with a high, intermediate and low probability of complete necrosis, was created. This scoring system enables early identification of the efficacy of TACE.

**Abstract:**

Transarterial chemoembolization (TACE) is used as a bridging treatment in liver transplant candidates with hepatocellular carcinoma (HCC). Alpha-fetoprotein (AFP) is the main tumor marker used for HCC surveillance. The aim of this study was to assess the potential of using the AFP change after the first TACE in the prediction of complete tumor necrosis. The study comprised 101 patients with HCC who underwent liver transplantation (LT) after TACE in the period between January 2011 and December 2020. The ΔAFP was defined as the difference between the AFP value before the first TACE and AFP either before the second TACE or the LT. The receiver operator characteristics (ROC) curves were used to identify an optimal cut-off value. Complete tumor necrosis was found in 26.1% (18 of 69) and 6.3% (2 of 32) of patients with an initial AFP level under and over 100 ng/mL, respectively (*p* = 0.020). The optimal cut-off value of ΔAFP for the prediction of complete necrosis was a decline of ≥10.2 ng/mL and ≥340.5 ng/mL in the corresponding subgroups. Complete tumor necrosis rates were: 62.5% (5 of 8) in patients with an initial AFP < 100 ng/mL and decline of ≥10.2 ng/mL; 21.3% (13 of 61) in patients with an initial AFP < 100 ng/mL and decline of <10.2 ng/mL; 16.7% (2 of 12) in patients with an initial AFP > 100 ng/mL and decline of ≥340.5 ng/mL; and null in 20 patients with an initial AFP > 100 ng/mL and decline of <340.5 ng/mL, respectively (*p* = 0.003). The simple scoring system, based on the initial AFP and AFP decline after the first treatment, distinguished between a high, intermediate and low probability of complete necrosis, with an area under the ROC curve of 0.699 (95% confidence intervals 0.577 to 0.821, *p* = 0.001). Combining the initial AFP with its change after the first treatment enables early identification of the efficacy of TACE.

## 1. Introduction

Hepatocellular carcinoma (HCC) is the sixth most common malignancy worldwide and the second cause of cancer-related deaths [1,2,3]. Liver transplantation is the most efficient treatment modality, enabling curative management of both cancer and the underlying liver pathology, which is the backbone of its development. Statistically, roughly 75% of patients can be cured with LT, compared to 25% to 40% after liver resection [4,5]. The Milan criteria, published by Mazzaferro in 1996, still serve as a benchmark for most liver transplant centers and they are recommended by international guidelines [6,7,8]. 

However, the Milan criteria are based solely on morphological features and do not comprise any factors addressing a tumor’s biology. Multiple studies have proven that these criteria are too restrictive; thus, a potentially curative approach cannot be offered to a significant number of candidates [9]. Nowadays, the refined approach mostly combines morphological parameters (number and size of tumors) and alpha-fetoprotein (AFP) concentration, which is a well-known surrogate of tumor biology. Accordingly, AFP concentration plays a pivotal role in currently accepted extended transplant criteria, which was elucidated in many studies, including, e.g., Mazzaferro et al. (Metroticket 2.0), Duvoux et al. (French AFP-model), Grąt et al. (Warsaw Criteria) and others. Published outcomes justify the implementation of these extended criteria. It is important from an ethical standpoint, as organ shortages are an ongoing issue [10,11,12,13,14]. 

Locoregional therapy (LRT) is often implemented as a bridging therapy aimed at local control of tumors while recipients await transplantation. This strategy may potentially decrease the drop-out rate, especially in patients with larger tumors or in regions struggling with organ shortages [15]. Additionally, patients that initially present with HCC beyond the Milan criteria may still be considered for transplantation if successful downstaging can be achieved [16,17]. A meta-analysis by Di Martino et al. revealed that from the intention-to-treat (ITT) analysis, long-term outcomes of patients after successful downstaging are similar to those initially within the listing criteria. Liver transplantation in this population provides much better outcomes compared with non-transplanted patients, which highlights the importance of LRT in this group [16]. 

Transarterial chemoembolization (TACE) is the most popular technique to help reach these goals [7,8,17]. Successful downstaging is perceived to be a favorable prognostic factor and yields very good long-term outcomes [17,18]. Efficient pre-transplant local therapy may lead to a significant response, including the absence of viable cancer cells in explanted livers (complete pathologic response—CPR). CPR can be achieved in up to 25% of patients, which translates into better long-term results in this group [19]. However, caution is required in the application of LRT, as a non-complete response may paradoxically increase the risk of recurrence compared with a treatment-naive group [20]. Changes in treatment-related AFP concentration is a well-known indicator of the response to therapy and a factor affecting long-term prognosis [21]. Despite effective downstaging, for many patients with multinodular HCC confined to the liver, LT is not an option for various reasons. Based on the well-recognized and the most popular system in the Western Hemisphere, the Barcelona Clinic Liver Cancer (BCLC) staging system, TACE is a definitive therapy in this population, with expected survival exceeding 2.5 years [22]. Similarly, other societies recommend TACE as a first-line treatment in patients with unresectable, non-transplantable multifocal HCC [23]. The aim of this study was to assess the role of AFP changes after the first TACE in the prediction of complete tumor necrosis in patients with HCC. 

## 2. Material and Methods

This retrospective study was based on a cohort of 272 patients with HCC who underwent LT in the Department of General, Transplant and Liver Surgery at the Medical University of Warsaw, including 135 patients who underwent TACE before surgery between January 2011 and December 2020. The study protocol was approved by the Medical University of Warsaw’s Human Research Committee. Due to the retrospective nature of the study, informed consent was not required. Patients with fibrolamellar HCCs, or combined hepatocellular/cholangiocellular carcinomas, patients who underwent TACE in other hospitals and those with missing data regarding AFP concentration and tumor necrosis were excluded. Finally, the study comprised 101 HCC patients after at least one TACE procedure prior to LT. A CPR after TACE was defined as an absence of viable tumor cells in the explanted liver, based on the pathology report. All histopathological examinations were carried out by a group of dedicated hepatobiliary pathologists. TACE using the combination of doxorubicin and lipiodol delivered via the femoral artery was a preferred approach. Two or three procedures with 4- to 8-week intervals, followed by radiological assessment, were scheduled a priori for all patients. Computed tomography (CT) and/or magnetic resonance imaging (MRI) were both allowed for the visualization of treatment efficacy. Additional TACE sessions were allowed if clinically indicated. All patients with HCC awaiting liver transplantation were discussed by a dedicated multidisciplinary tumor board comprised of a liver transplant surgeon, radiologist, hepatologist and oncologist. Indications for neoadjuvant treatment were decided individually based on the liver function, clinical staging and risk of progression with a potential subsequent drop-out from the transplant list. The study cohort was divided into two subgroups based on the AFP < 100 ng/mL and AFP > 100 ng/mL before the first TACE. The AFP threshold was chosen following the previously published results [12]. The optimal ΔAFP was estimated independently for both groups. The ΔAFP was calculated as the difference between the AFP value before the first TACE and AFP either before the second TACE or the LT. The receiver operator characteristics (ROC) curves were used to identify an optimal cut-off point for ΔAFP. Then, four groups were created: AFP < 100 ng/mL and ΔAFP greater (group 1) and lower than the cut-off point (group 2). Similarly, an additional two groups with AFP > 100 ng/mL and ΔAFP greater (group 3) and lower than the cut-off point (group 4) were established. Subsequently, groups 2 and 3 were merged together. Following this, a score based on the dynamics of AFP after the first TACE with a low, intermediate and high probability of CPR was calculated based on the remaining three groups. The primary objective of the study was to find a correlation between AFP dynamics after the first TACE and CPR. Quantitative and qualitative data were presented, respectively, as medians with interquartile ranges and as numbers with frequencies. The chi-square test was used to compare subgroups. Logistic regression was used to assess a certain AFP concentration as a predictor of CPR. Odds ratios (ORs) and areas under the curve (AUCs) were presented with 95% confidence intervals. The level of significance was set at *p* < 0.05. Statistical analyses were computed in STATISTICA version 13.3 (TIBCO Software Inc., Palo Alto, CA, USA). 

## 3. Results

**Figure 1:**
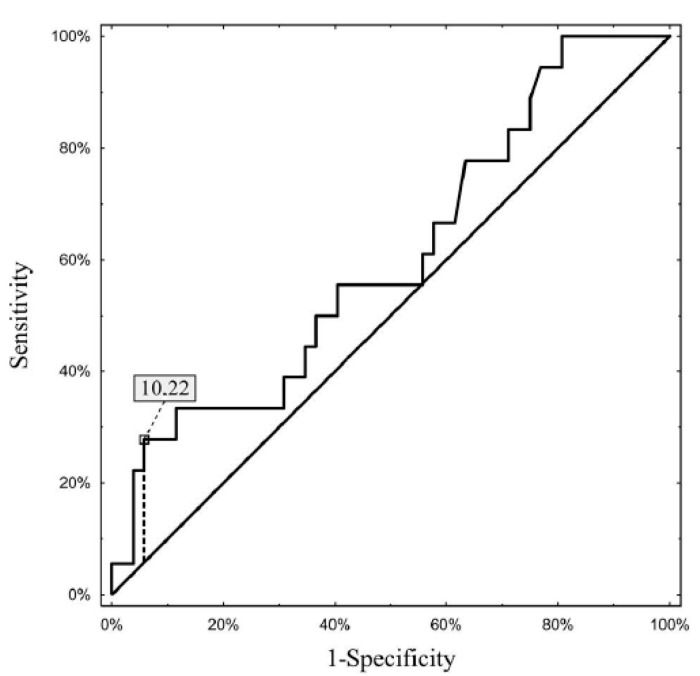
Optimal ΔAFP in the subgroup with AFP < 100 ng/mL in prediction of complete tumor necrosis.

**Figure 2:**
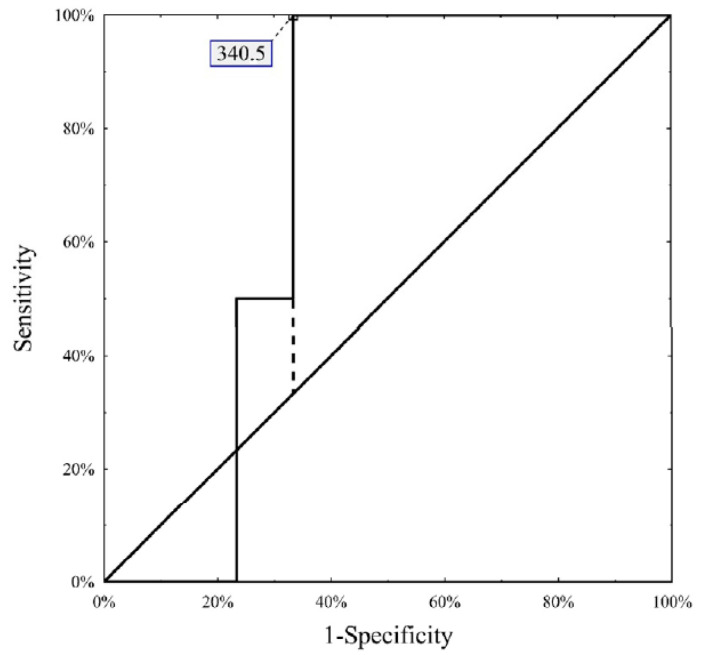
Optimal ΔAFP in the subgroup with AFP > 100 ng/mL in prediction of complete tumor necrosis.

In the study cohort, males dominated (79 of 101, 78.2%). The median MELD score was 9 (range 6–22) and 69 patients (68.3%) had an AFP concentration < 100 ng/mL before the first TACE. The median number of tumors was one (range 1–10) and the median number of TACE procedures was two (range 1–6). The Milan criteria were fulfilled by 61.9% of patients. In the whole analyzed group, CPR was observed in 20 patients (19.8%). Detailed characteristics of the study cohort are presented in Table 1. An initial AFP level < 100 ng/mL increased the probability of CPR (OR 5.294, 95% confidence intervals 1.15 to 24.42, *p* = 0.033) compared with an initial AFP > 100 ng/mL. Complete tumor necrosis was found in 26.1% (18 of 69) and 6.3% (2 of 32) of patients with an initial AFP under and over 100 ng/mL, respectively (*p* = 0.020). In the subgroup with an AFP < 100 ng/mL, a prediction of complete tumor necrosis based on ΔAFP was associated with an AUC of 0.604 (95% confidence intervals 0.452 to 0.756, *p* = 0.180) and an optimal cut-off of ≥10.2 ng/mL (Figure 1). In the group with an initial AFP level > 100 ng/mL, a prediction of complete tumor necrosis based on ΔAFP was associated with an AUC of 0.717 (95% confidence intervals 0.546–0.887, *p* = 0.013) and an optimal cut-off of ≥340.5 ng/mL (Figure 2). Complete tumor necrosis rates were: 62.5% (5 of 8) in patients with an initial AFP level < 100 ng/mL and decline of ≥10.2 ng/mL (group 1); 21.3% (13 of 61) in patients with an initial AFP level < 100 ng/mL and decline of <10.2 ng/mL (group 2); 16.7% (2 of 12) in patients with an initial AFP level > 100 ng/mL and decline of ≥340.5 ng/mL (group 3); and null in 20 patients with an initial AFP level > 100 ng/mL and decline of <340.5 ng/mL (group 4), respectively (*p* = 0.003). A simple score based on the initial AFP level and AFP decline after the first treatment distinguished between a high (group 1), intermediate (groups 2 and 3) and low (group 4) probability of complete necrosis with an area under the ROC curve of 0.699 (95% confidence intervals 0.577 to 0.821, *p* = 0.001).

## 4. Discussion

The response to LRT treatment, measured by the decrease in tumor burden with a concomitant decline of AFP levels or its low initial concentration, is a surrogate of less aggressive tumor biology, which thus yields a good long-term prognosis after liver transplantation, with 5-year survival rates reaching 70% [23]. The recently published large series of patients with HCC clearly underlines the crucial role of downstaging in patients initially beyond the benchmark transplant criteria. A study by Tabrizian et al. comprising over 2500 patients with HCC showed excellent results with 5- and 10-year survival rates as high as 67.9% and 52.1%, respectively with median survival over 120 months and a 15% recurrence rate among patients who were successfully downstaged to the Milan criteria [18]. Nevertheless, the application of LRT carries a risk for these patients in whom downstaging cannot be ultimately achieved. Data from the US corroborates this observation. Kardashian et al. revealed that patients beyond the Milan criteria who were not able to achieve a satisfactory response had a greater risk of HCC recurrence compared with the treatment-naïve group (34.1% vs. 26.1%; *p* < 0.001 [24]). This observation is postulated by other authors as well, which makes effective LRT a crucial marker of more indolent tumor biology. Thus, it appears to be one of the major drivers determining the ultimate results of treatment in patients with advanced HCC listed for LT [9,16,17,18]. One explanation is that unsuccessful LRT just unmasks tumors with more favorable biology. However, local tissue changes after LRT, with the subsequent creation of a microenvironment promoting cancer cells’ growth, is a phenomenon which needs further investigation as well [24]. 

Similarly, non-complete pathologic response after LRT was found to negatively affect long-term outcomes in patients with HCC after liver transplantation. A study from the Medical University of Warsaw found that non-complete CPR is linked to a higher risk of recurrence in an otherwise low-risk group, including patients within the Milan and Warsaw criteria and those with a maximum of 2 points according to the AFP model [20]. Herein, we present a simple score aimed at forecasting the CPR in patients with HCC awaiting liver transplantation. Based on the initial AFP concentration and its dynamic changes after the first TACE, we were able to identify groups with different probabilities of CPR with satisfactory accuracy (AUC 0.699). We divided our cohort into two subgroups using a cut-off value of the initial AFP level set at 100 ng/mL and analyzed them separately. This was supported by our previous study, which underlined a markedly different prognosis in patients with AFP lower or greater than the chosen threshold. In the study by Grąt et al., the excellent outcomes with no recurrence observed in patients transplanted with a last pre-transplant AFP level < 100 ng/mL, despite presenting advanced tumors beyond the Milan criteria, are highlighted [12]. Similarly, Mehta et al. analyzed almost 4000 patients from the United Network for Organ Sharing (UNOS) database. Among those within the extended criteria, meeting either UNOS-downstaging (UNOS-DS) or “all-comers” (AC-DS) criteria, the last pre-transplant AFP level > 100 ng/mL was found to be the only predictive factor negatively related to HCC recurrence. Recurrence was observed in 26% and 12.7% of patients with AFP levels > 100 ng/mL and <100 ng/mL, respectively (*p* = 0.03 [25]). The prognostic importance of this cut-off AFP value in patients with HCC was confirmed by other authors as well [11]. In the present study, the probability of achieving CPR was higher among patients with an initial AFP level < 100 ng/mL, compared with the remaining patients, and this cut-off was found to be a significant predictor of CPR. Moreover, our analysis was able to identify a subgroup with >60% chance of CPR after the first TACE (initial AFP level < 100 ng/mL and decline of ≥10.2 ng/mL), as well as the group with the highest risk of failure, where CPR is highly unlikely (initial AFP level > 100 ng/mL and decline of <340.5 ng/mL). The ability to foresee the effect of LRT is crucial, as data show that only patients with CPR can actually benefit from this strategy [26,27]. In their study, Agopian et al. found that patients who achieved CPR had the longest 5-year recurrence-free survival (RFS) and the lowest rate of tumor recurrence. Interestingly, patients whose tumors were not eradicated completely (no-CPR group) experienced an over 30% higher risk of HCC recurrence compared with those with no LRT applied [27]. Furthermore, increasing the number of TACE procedures before transplantation portends worse outcomes with a lower probability of achieving CPR and a higher risk of HCC recurrence [26,27]. Similarly, a multicenter study from Europe analyzing the role of LRT in patients within the Milan criteria, showed that, in the ITT analysis, the highest protection against HCC-dependent failure was linked with only one treatment session, and when ≥4 treatments were needed, the protective effect of LRT was lost [28]. Data regarding the significant role of the dynamic changes of AFP following TACE were published previously not only in liver transplant patients but also in those where LRT was applied as a first-line treatment. In their study, Mishra et al. showed that patients with AFP < 200 ng/mL before and after the first TACE had a better prognosis compared with those with an initial AFP ≥ 200 ng/mL or progressing beyond this threshold after the first procedure (*p* = 0.0001, [29]). Similarly, other authors have also revealed a positive correlation between AFP response and better long-term results. However, in all studies, response to treatment was assessed using radiological studies [21,30]. Compared with others, the present study provides a unique perspective analyzing the correlation between AFP changes and CPR. Data show that computed tomography (CT) may overestimate the response to neoadjuvant treatment. Thus, microscopic evaluation of whole-explanted liver provides the ultimate and the most granular method of assessment of the efficacy of LRT [31]. 

Taken together, we provide strong evidence highlighting the role of AFP in risk stratification of patients with HCC undergoing LT. Due to the potentially detrimental impact of suboptimal LRT on long-term prognosis in this group, early identification of those with a low probability of complete response is of the utmost importance and has crucial clinical implications. Because unsuccessful LRT may jeopardize the ultimate results in patients within and beyond the Milan criteria, a management strategy should be verified and tailored accordingly. One of the solutions is prompt intensification of treatment, as data confirms that a second TACE yields comparable efficacy to the initial procedure, with a complete response experienced in roughly 50% of patients [32] Another approach is to combine thermal or microwave ablation with TACE. This combined strategy (TACE plus ablation) seems to be more efficient than TACE or ablation alone, providing greater efficacy in achieving CPR. A study by Agopian et al. revealed a 35% probability of CPR in the combined approach, compared with 26% after ablation and 19% after TACE were used as stand-alone modalities. Longer waiting times between LRT and LT, instead of an immediate listing, may additionally increase rates of CPR [19]. In cases where a lobar TACE is used, a selective or superselective approach should be applied, as this technique may also increase rates of tumor necrosis. In their study, Golfieri et al. described that selective or superselective TACE translated into greater mean levels of necrosis (75.1% versus 52.8% for lobular TACE, *p* = 0.002) and a significantly higher probability of complete necrosis (53.8% versus 29.8% for lobular TACE, *p* = 0.013). Nevertheless, additional TACE sessions were needed when the lobular approach was used [31]. Another interesting option refers to Y-90 radioembolization. Gabr et al. revealed that CPR can be experienced in 45% of patients. Very encouraging long-term results were observed with a median RFS as high as 120 months. More importantly, results were comparable between those within and beyond the Milan criteria, and the drop-out rate due to progression in patients bridging to LT was only 5.2% [33].

Our results can be interpreted, not only with regard to LT but in the context of the wider population of patients with HCC at different stages of disease treated by means of TACE. Good responses to LRT with a concomitant decrease in AFP concentration serves as an indicator of a favorable prognosis not only in patients listed for transplantation. A prospective study from Japan, analyzing over 8000 patients with unresectable HCC treated with TACE, highlighted a low AFP concentration as one of the significant risk factors of shorter survival [34]. Nevertheless, controversy still exists with regard to the heterogenous population of patients with multifocal non-transplantable disease, where TACE is recommended according to the BCLC staging system [22]. However, this approach is currently challenged, as liver resections can be offered to well-selected HCC patients with BCLC stage B/C disease, providing better outcomes compared with TACE [35,36]. Moreover, data from China show that preoperative TACE may improve the long-term results of liver resection, with the longest RFS observed in those with CPR after induction LRT [37]. Thus, our simple score may be used as an additional tool in the decision-making process in patients with HCC confined to the liver who are not liver transplant candidates.

This is a retrospective study with all its inherent limitations. It is a single-center study with a relatively small sample size. Despite some data being missing, relevant data related to AFP changes and full pathologic reports were analyzed in the whole cohort. Additionally, a precise correlation between AFP fluctuations after the initial TACE and CPR has never been reported according to the authors’ knowledge which, undoubtedly, is the strength of this study.

## 5. Conclusions

Combining initial AFP concentration with its dynamic changes after the first treatment enables early identification of the efficacy of TACE and may be helpful in optimal application of LRT in patients with HCC. 

## Figures and Tables

**Table 1:** Characteristics of the study cohort.

	Median (Range) or n (%)
Recipient age (years)	58 (27–70)
Recipient sex (male)	79 (78.2%)
BMI	27.4 (25.4–33.8)
HBV (n = 100)	42 (42%)
HCV (n = 99)	59 (59.6%)
MELD	9 (6–22)
AFP before first TACE < 100 (ng/mL)	69 (68.3%)
Previous liver resection (n = 88)	8 (9.1%)
Number of tumors	1.0 (1–10)
Size of the largest tumor (mm)	35 (7–140)
Number of TACEs	2 (1–6)
Microvascular invasion (n = 84)	25 (29.8%)
Grading G3 (n = 66)	8 (12.1%)
Milan criteria (n = 84)	52 (61.9%)
Up-to-7 criteria (n = 84)	61 (72.6%)
Complete pathologic response (whole cohort)	20 (19.8%)
Complete pathologic response AFP ≤ 100 (ng/mL, n = 69)	18 (26.1%)
Complete pathologic response AFP > 100 (ng/mL, n = 32)	2 (6.3%)
Data are presented as medians (range) or n (%)	

## Data Availability

The data presented in this study are available upon justified request.
